# Genetic Factors Control Nicotine Self-Administration in Isogenic Adolescent Rat Strains

**DOI:** 10.1371/journal.pone.0044234

**Published:** 2012-08-28

**Authors:** Hao Chen, Katie A. Hiler, Elizabeth A. Tolley, Shannon G. Matta, Burt M. Sharp

**Affiliations:** 1 Department of Pharmacology, University of Tennessee Health Science Center, Memphis, Tennessee, United States of America; 2 Department of Preventive Medicine, University of Tennessee Health Science Center, Memphis, Tennessee, United States of America; Centre for Addiction and Mental Health, Canada

## Abstract

Adult cigarette smokers usually become dependent on cigarettes during adolescence. Despite recent advances in addiction genetics, little data delineates the genetic factors that account for the vulnerability of humans to smoke tobacco. We studied the operant nicotine self-administration (SA) behavior of six inbred strains of adolescent male rats (Fisher 344, Brown Norway, Dark Agouti, Spontaneous Hypertensive Rat, Wistar Kyoto and Lewis) and six selected F1 hybrids. All rats were trained to press a lever to obtain food starting on postnatal day (PN) 32, and then nicotine (0.03 mg/kg/infusion, i.v.) reinforcement was made available on PN41-42 (10 consecutive daily 2 h sessions). Of the 12 isogenic strains, Fisher rats self-administered the fewest nicotine infusions (1.45±0.36/d) during the last 3 d, while Lewis rats took the most nicotine (13.0±1.4/d). These strains sorted into high, intermediate and low self-administration groups in 2, 2, and 8 strains, respectively. The influence of heredity on nicotine SA (0.64) is similar to that reported for humans. Therefore, this panel of isogenic rat strains effectively models the overall impact of genetics on the vulnerability to acquire nicotine-reinforced behavior during adolescence. Separate groups of rats responded for food starting on PN41. The correlation between nicotine and food reward was not significant. Hence, the genetic control of the motivation to obtain nicotine is distinctly different from food reward, indicating the specificity of the underlying genetic mechanisms. Lastly, the behavior of F1 hybrids was not predicted from the additive behavior of the parental strains, indicating the impact of significant gene-gene interactions on the susceptibility to nicotine reward. Taken together, the behavioral characteristics of this model indicate its strong potential to identify specific genes mediating the human vulnerability to smoke cigarettes.

## Introduction

Tobacco use is the single most preventable cause of disease, disability, and death in the United States. Approximately 20% of US adults smoke cigarettes. Each year, approximately 443,000 premature deaths are attributable to smoking or exposure to second hand smoke. Among adult smokers, 85% began before age 21 and 68% prior to 18 [1], [2], making adolescence the critical stage for initiation of smoking. Despite these dismal statistics, our knowledge of the environmental and genetic factors that predispose adolescents to initiate and maintain tobacco smoking is very limited.

Nicotine is the principal psychoactive ingredient of tobacco products. The effect of nicotine on motivated behavior (i.e., wanting and using cigarettes) is often modeled using operant self-administration (SA) procedures. This model pertains to a variety of species, such as mice, rats, primates and even human. Amongst these, the rat is the most widely studied, and a variety of nicotine-modulated behaviors have been demonstrated, such as dependence, withdrawal, extinction, and relapse [3]–[6]. In adolescent rats, we found [7] more rapid acquisition of nicotine self-administration, reaching higher intake levels, than adults.

The overall impact of genetic and genomic differences on the vulnerability to smoke has been estimated at ∼ 0.5 in numerous heritability studies [8], [9]. However, very few specific risk genes have been identified. Recent genome-wide studies confirmed the association of genetic variation in the CHRNA5-CHRNA3-CHRNB4 gene cluster with smoking phenotypes [10]–[12]. A few other candidate genes also have been studied. However, the contribution of these genes is quite small. For example, within CHRNA3, the single nucleotide polymorphism (SNP) with the greatest association to smoking accounted for only 0.5–0.7% of the variance to smoke [10]–[12]. In all probability, many other unidentified genes contribute to the vulnerability to smoke.

Rodent models can unambiguously identify candidate genes because both genetic and environmental factors are controllable. The success of such a model requires evidence of strong phenotypic variation in a smoking-specific behavior. This can be accomplished by identifying a panel of inbred rodent strains that differ in their nicotine SA behavior. More than 500 strains of inbred rats have been described [13], and the genome sequences of two of them [14], [15] are known, However, operant nicotine self-administration has not been reported for most of these strains. Therefore, we bred a panel of six adolescent inbred rat strains and six selected F1 adolescent offspring in order to test the hypothesis that inbred genetic differences determine nicotine self administration during adolescence. Experiments reported herein demonstrate very strong behavioral variation across these adolescent inbred strains and isogenic F1 crosses.

## Results

### Nicotine SA

**Figure 1:**
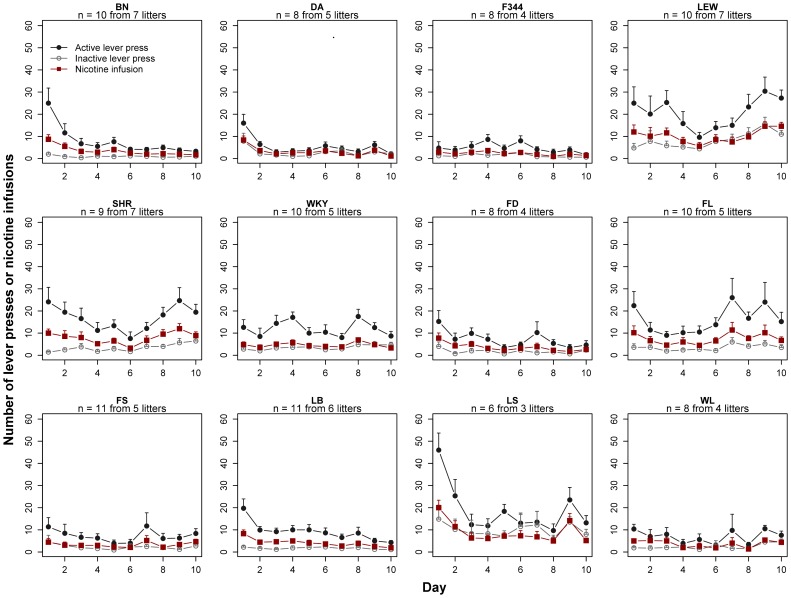
Nicotine self-administration by isogenic male adolescent rats. Six inbred and six isogenic F1 hybrid strains were trained to press a lever for food on postnatal day 33. Nicotine SA started on postnatal day 41 or 42. Nicotine (30 µg/kg/infusion, i.v.) was delivered using a fixed-ratio 1 schedule. Each session lasted 2 h. A total of 10 daily sessions were conducted without interruption. Statistical analyses of the number of lever presses and nicotine infusions for the last 3 d were shown in Table 1. BN: Brown Norway; DA: Dark Agouti; F344: Fisher 344; LEW: Lewis; SHR: Spontaneous hypertensive rat; WKY: Wistar Kyoto. F1 hybrids were identified by the initials of the parental strains.

**Figure 2:**
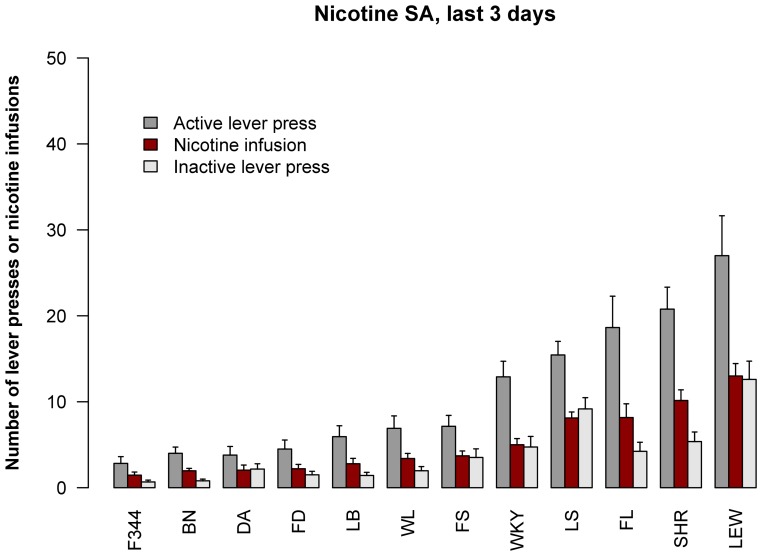
Strain differences in nicotine self-administration. The means for the number of active and inactive lever presses as well as nicotine infusions are shown for the last 3 d of SA. Strains are ordered by the number of nicotine infusions. Results for pair-wise comparisons are shown in Table 2.

For each rat strain, the numbers of active v. inactive lever presses during the entire 10 d of nicotine SA are shown in Figure 1. Due to prior food training, active lever presses during the first few days of nicotine SA were most likely motivated, in part, by the drive to obtain food. This is evident in the transient elevation of active lever presses during the first 1–3 d in many strains (e.g., LEW, BN, LB, LS). Therefore, we analyzed the number of lever presses and nicotine infusions during the last 3 d of SA (Figure 2) using repeated measures ANOVA. The effect of Day was significant only for two strains, both showing low nicotine intake [DA (F_2,14_ = 8.57, p<0.01) and WL (F_2,14_ = 13.08, p<0.01)]. All strains showed a significant main effect of Lever, with the exception of DA (statistics shown in Table 1). We have previously reported that F344 did not show significant lever preference using a 23 hr access model [16]. This discrepancy is most likely due to the food training utilized in the present study. No significant interaction between Day and Lever was found with the exception of WKY (F2,18 = 4,7, p<0.05). Overall, these data showed that this panel of isogenic rats established stable nicotine SA, as demonstrated by the lack of day-to-day variation and strong preference for the active lever (excepting DA rats).

**Table 1:** Statistics on the effect of lever in each strain during the last 3 d of nicotine and food SA.

	Nicotine			Food		
Strain	Df	F	p	Df	F	p
BN	(1,9)	22.04	***0.001***	(1,8)	168.50	***<0.001***
DA	(1,7)	2.00	0.200	(1,7)	86.85	***<0.001***
F344	(1,7)	8.70	***0.021***	(1,10)	44.92	***<0.001***
LEW	(1,9)	16.53	***0.003***	(1,8)	100.20	***<0.001***
SHR	(1,8)	82.99	***<0.001***	(1,6)	164.90	***<0.001***
WKY	(1,9)	18.78	***0.002***	(1,8)	46.36	***<0.001***
FD F1	(1,7)	10.70	***0.014***	(1,9)	96.36	***<0.001***
FL F1	(1,9)	23.73	***0.001***	(1,9)	50.45	***<0.001***
FS F1	(1,10)	14.40	***0.004***	(1,8)	29.34	***0.001***
LB F1	(1,10)	13.56	***0.004***	(1,8)	117.50	***<0.001***
LS F1	(1,5)	7.83	***0.038***	(1,7)	120.50	***<0.001***
WL F1	(1,7)	9.93	***0.016***	(1,9)	120.40	***<0.001***

Active v. inactive lever presses (numbers not shown) during the last 3 d of nicotine or food SA were analyzed using repeated measures ANOVA for each isogenic strain. The p values that achieved statistical significance (p<0.05) are highlighted in bold and italics.

Of these strains, F344 self-administered the fewest nicotine per day (1.45±0.36 infusions), while LEW took the most (13.0±1.4) – an 8.9-fold difference. Pair-wise comparisons between strains in the number of nicotine infusions are listed in Table 2. F344 was not significantly different from BN, DA, WKY or FD, FS, LB and WL. Thus, these strains are the low nicotine SA cohort. LEW rats were not significantly different from SHR; these 2 strains are the high nicotine SA cohort. Only FL and LS, the median cohort, were significantly different from both high and low cohorts.

**Table 2:** Nicotine infusion during the last 3 d of SA (Tukey HSD adjusted pair-wise p values).

	BN	DA	F344	FD	FL	FS	LEW	LB	LS	SHR	WKY
**DA**	1.000										
**F344**	1.000	1.000									
**FD**	1.000	1.000	1.000								
**FL**	***<0.001***	***0.001***	***<0.001***	***0.001***							
**FS**	0.989	0.996	0.934	0.999	***0.007***						
**LEW**	***<0.001***	***<0.001***	***<0.001***	***<0.001***	***0.008***	***<0.001***					
**LB**	1.000	1.000	0.996	1.000	***0.001***	1.000	***<0.001***				
**LS**	***0.002***	***0.005***	***0.001***	***0.007***	1.000	***0.046***	***0.039***	***0.012***			
**SHR**	***<0.001***	***<0.001***	***<0.001***	***<0.001***	0.917	***<0.001***	0.516	***<0.001***	0.960		
**WKY**	0.375	0.508	0.236	0.598	0.310	0.972	***<0.001***	0.793	0.562	***0.005***	
**WL**	0.999	1.000	0.987	1.000	***0.004***	1.000	***<0.001***	1.000	***0.026***	***<0.001***	0.906

The mean number of nicotine infusions taken by each strain during the last 3 d of SA was compared using a post-hoc Tukey HSD procedure. The p values are shown for all the pair-wise comparisons. Comparisons that achieved statistical significance (p<0.05) are in bold and italics.

### Food rewarded behavior

**Figure 3:**
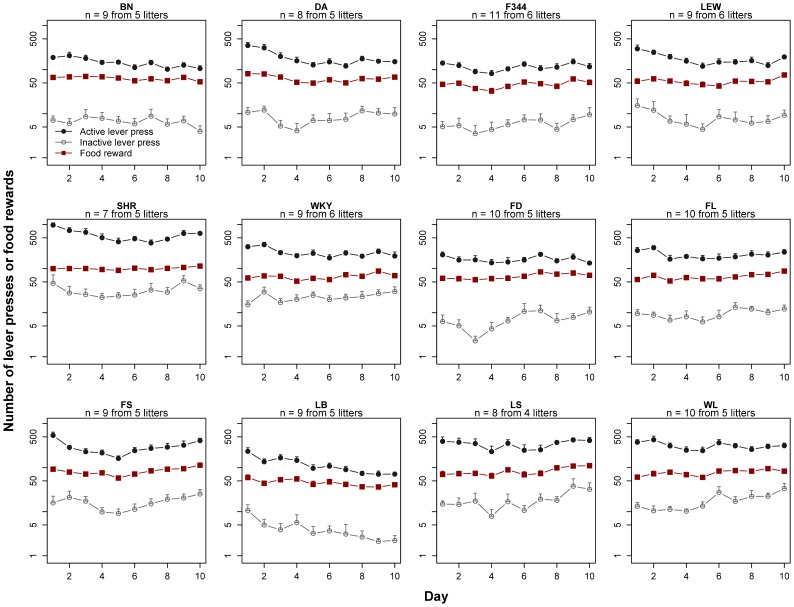
Food rewarded behavior by isogenic male adolescent rats. Six inbred and six isogenic F1 hybrid strains were trained to press a lever for food on postnatal day 33–36. Food reward resumed on postnatal day 41 or 42. Food pellets were delivered using a fixed-ratio 1 schedule. Each session lasted 2 h. A total of 10 daily sessions were conducted without interruption. Statistical analyses of the number of lever presses and food rewards earned are shown in Table 1. (Y-axis is logarithmic).

**Figure 4:**
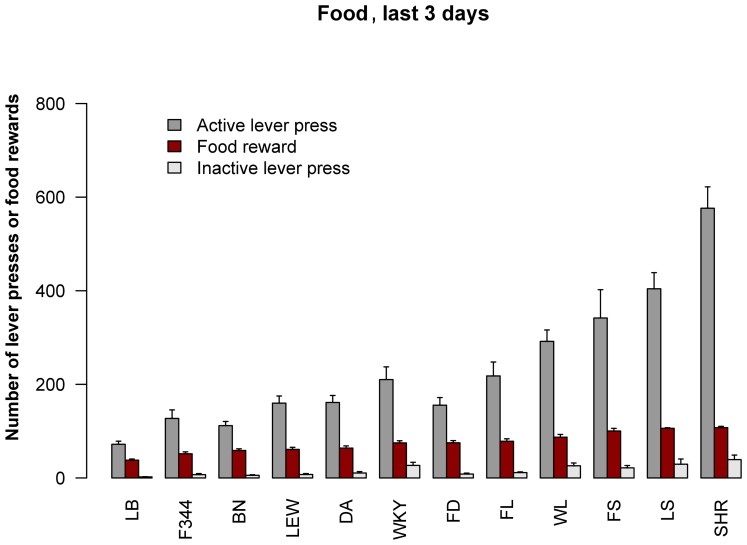
Strain differences in food rewarded behavior. The means for the number of active and inactive lever presses, as well as food reward earned, are shown for the last 3 d of SA. Strains are ordered by the number of food rewards earned. Results for pair-wise comparisons are shown in Table 3.

For each rat strain, the numbers of active vs. inactive lever presses during the entire 10 days of food SA are shown in Figure 3. As for nicotine SA, we analyzed the number of food rewards obtained by strain during the last 3 d. No significant effect of Day was found for any strain, nor was there any significant Day and Lever interaction. All strains demonstrated significant preference for the active lever (statistics shown in Table 1). Figure 4 indicates that LB F1 received the least food per day (38.0±2.4 pellets), while SHR obtained the most (107.5±2.8) – a 2.8-fold difference.

Pair-wise comparisons between strains in the number of food pellets obtained during the last 3 d are shown in Table 3. LB rats were not different from BN and F344; these are the low cohort. The high cohort consists of SHR, FS and LS. The remaining strains (LEW, DA, WKY, FD, FL and WL) comprise the median group.

**Table:** **Table 3.** Strain differences in food rewards earned during the last 3 d of SA.

	BN	DA	F344	FD	FL	FS	LEW	LB	LS	SHR	WKY
**DA**	1.000										
**F344**	0.990	0.728									
**FD**	0.271	0.833	***0.007***								
**FL**	0.081	0.501	***0.001***	1.000							
**FS**	***<0.001***	***<0.001***	***<0.001***	***0.005***	***0.029***						
**LEW**	1.000	1.000	0.925	0.496	0.193	***<0.001***					
**LB**	0.064	***0.008***	0.520	***<0.001***	***<0.001***	***<0.001***	***0.023***				
**LS**	***<0.001***	***<0.001***	***<0.001***	***<0.001***	***0.002***	0.999	***<0.001***	***<0.001***			
**SHR**	***<0.001***	***<0.001***	***<0.001***	***<0.001***	***0.002***	0.996	***<0.001***	***<0.001***	1.000		
**WKY**	0.330	0.870	***0.011***	1.000	1.000	***0.007***	0.564	***<0.001***	***<0.001***	***<0.001***	
**WL**	***0.001***	***0.021***	***<0.001***	0.702	0.951	0.604	***0.003***	***<0.001***	0.138	0.106	0.711

The mean number of food pellets earned by each strain during the last 3 d of SA was compared using a post-hoc Tukey HSD procedure. The p values are shown for all the pair-wise comparisons. Comparisons that achieved statistical significance (p<0.05) are in bold and italics.

### Correlation between food rewarded behavior and nicotine SA

**Figure 5:**
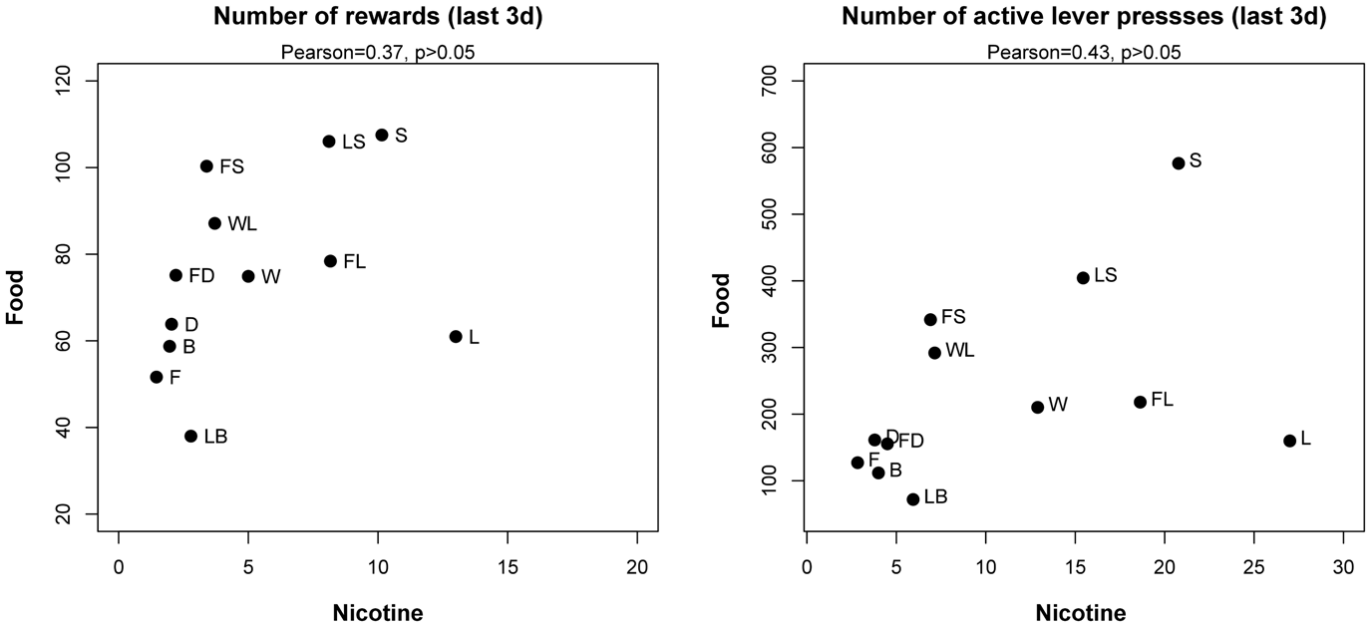
Correlation between nicotine SA and food reward. The numbers of nicotine and food rewards earned by the 12 strains during the last 3 days were not statistically significant. The correlation of active lever presses during nicotine v. food SA was not significant.

The mean number of active lever presses and rewards earned during the last 3 d by rats self-administering nicotine vs. obtaining food reward were compared across all strains (Fig. 5). Pearson correlation coefficients were 0.43 and 0.37, respectively, and neither was statistically significant.

### Genetic effects on nicotine SA and food rewarded behavior

The narrow-sense heritability for nicotine SA, calculated based on the mean nicotine intake during the last 3 d of SA, was 0.64, while the heritability for food reward was 0.71. The difference between F1 and the expected means of the two parental strains are listed in Table 4 for both nicotine intake and food reward. For nicotine SA, four (LB, FL, LS, FS) of the six F1 crosses significantly differed from the expected additive genetic effects of the parental strains. For food SA, all six F1 crosses were significantly different from the expected additive genetic effects of the parental strains.

**Table 4:** Difference between F1 and the mean expected from the parental strains.

Strain	Nicotine intake	Food Reward
LB v. LEW & BN	***<0.001***	***<0.001***
FD v. F344 & DA	0.794	*0.001*
FL v. F344 & LEW	***<0.001***	***<0.001***
WL v. WKY & LEW	0.468	***0.001***
LS v. LEW & SHR	***0.008***	***<0.001***
FS v. F344 & SHR	***0.018***	***<0.001***

The amount of nicotine intake or food reward earned by each F1 hybrid strain during the last 3 d was compared to the mean expected from the two parental strains. This value for the mean of the two parental strains predicts F1 behaviors if the genetic determinants were additive. Comparisons that achieved statistical significance (p<0.05) are in bold and italics.

## Discussion

To develop an animal behavioral model capable of elucidating the role of genetics in the vulnerability to smoke cigarettes during adolescence, we focused on nicotine, the principal psychoactive agent in tobacco smoke. We found that nicotine-reinforced operant behavior varies significantly across a panel of 12 isogenic strains of rats in mid-to-late adolescence. These included 6 unique F1 hybrids. These 12 strains displayed a full spectrum of motivated nicotine intake, ranging from an average of 1.4 to 13.0 infusions per 2 h session. This variation in behavior across isogenic strains was largely due to inheritance (0.64). We also found no correlation between motivated nicotine SA and food reward, indicating that genetic control of the motivation to obtain nicotine is distinct from natural rewards.

Genetics plays a major role in the susceptibility to substance abuse and addictive disorders [9], [17]. Human studies [8], [9] have reported a heritability of about 0.5 for various cigarette-smoking phenotypes (e.g., cigarettes per day). This is similar to the estimated heritability of nicotine intake found in our study (0.64), indicating that genetic mechanisms influence the vulnerability to abuse nicotine to a similar degree in both rodents and humans. Despite the large effort involved in several recent genome-wide association studies, few specific risk genes have been identified. One of them is the CHRNA5-CHRNA3-CHRNB4 gene cluster located on chromosome 15q24-25 [10]–[12]. The contribution of several other candidate genes to the vulnerability to cigarette-smoking has been reported, such as catechol O-methyltransferase [18], dopamine receptor 2 [18]–[21], opioid receptor [22] as well as genes related to nicotine metabolism, such as cytochrome P450 CYP 2A6 [23], [24]. The effects of these genes are often dependent on the ethnicity of the population [18], [25], [26]. In general, the contribution of each gene is quite small, consistent with the notion that smoking is a complex trait determined by many genetic loci.

The inability to control for myriad environmental variables is one of the foremost limitations in detecting the genetic loci that determine the human vulnerability to smoke cigarettes. Rodent models circumvent these limitations, especially when a dedicated team breeds and evaluates all strains in the same facility to avoid inadvertent stressors, such as those due to shipping. Although mice have traditionally been the principal model for genetic studies, establishing nicotine SA in mice is fraught with difficulties. These include not only technical difficulties (e.g., implanting and maintaining a chronically, patent indwelling catheter), but more critically, the challenge of unambiguously attributing the observed behavior directly to i.v. nicotine [27]. For example, the number of self-administered nicotine infusions appear to be similar [28] to the number of saline infusions obtained by control mice (but see [29]). In contrast, established rat models of nicotine SA consistently surmount these limitations [3]–[5].

The recent availability of rat genomic resources enables genetic studies of rat behavior. For example, the genome sequence of both BN [14] and SHR [15] strains have been published, and the sequences of many other rat strains (currently 17) are also available (http://rgd.mcw.edu). Additionally, single nucleotide polymorphisms, comprising ∼20,000 locations, are available for 167 strains of rat [30]. These resources provide the foundation for behavioral genetic studies of inbred and F1 rats.

This study identified a large difference in stable nicotine intake among isogenic strains, with F344 and LEW strains at the extreme ends of the spectrum, respectively. Although the current study used a 2 h limited access model of nicotine SA, the contrasting behavior of the LEW and F344 strains is in agreement with our previous findings obtained from a 23 h model of virtually unlimited access to nicotine SA [16]. In fact, these two strains are the most frequently studied inbred strains in models of drug addiction. In general, LEW rats self-administer more morphine than F344 rats [31]–[34] but F344 rats self-administer more cocaine than LEW rats [35]–[37]. However, the precise hierarchy of strain-dependent nicotine SA reported herein may be affected by unknown differences in the dose-response profiles between strains, such that nicotine 30 µg/kg might be on the descending limb in some strains. Our finding that food maintained behavior was not significantly different between these two strains (Table 4) is consistent with our previous report [48]. Taken together, these results suggest that not only are the genetic mechanisms underlying natural vs. drug-reinforced behavior different, but the genetic control of drug abuse is substance-specific.

In the present studies, F1 hybrids were used to identify additional complexity in the genetic control of nicotine-reinforced operant behavior. Indeed, in four of the F1 crosses, the amount of nicotine intake was different from the expected additive genetic effects of the parental strains (Table 4). This is consistent with the impact of specific gene-gene interactions on a complex trait like nicotine SA.

In summary, we developed a unique panel of isogenic rat strains that effectively model the overall impact of genetics on the vulnerability to acquire nicotine-reinforced behavior during adolescence. The influence of heredity on this process (h^2^ = 0.64) is similar to that reported for humans. Moreover, in this model, the genetic control of the motivation to obtain nicotine is distinctly different from food reward, indicating the specificity of the underlying genetic mechanisms. Significant gene-gene interactions were found to determine the susceptibility to abuse nicotine in any particular rat strain, as shown by the failure to accurately predict F1 behavior based simply on the inheritance of additive genetic factors from the parental strains. Taken together, these characteristics of the model indicate its strong potential to identify specific genes mediating the human vulnerability to smoke cigarettes, a problem that is exceedingly difficult to resolve by human studies alone.

## Materials and Methods

### Ethics Statement

All procedures were conducted in accordance with the NIH Guidelines concerning the Care and Use of Laboratory Animals, as approved by the Animal Care and Use Committee of the University of Tennessee Health Science Center. Ketoprofen (2 mg/kg, s.c., administered once) was given for post-operative analgesia.

### Animals

Breeders for six inbred rat strains, including BN, SHR, Dark Agouti (DA), Wistar Kyoto (WKY), Lewis (LEW), and Fisher 344 (F344) were obtained from Harlan Laboratories (Indianapolis, IN). Rats were housed in a 12∶12 h reversed light cycle (lights off at 10∶30 h) with food and water available *ad libitum*. Inbreeding was maintained within each strain. Six selected F1 hybrids were also generated, including LB, FL, FS, WL, FD, LS (the two letters represent the initials of the maternal and paternal strains, respectively).

For SA, all animals were bred in our animal facility, thereby eliminating the potentially confounding effect of shipping stress on behavior. Only male adolescent offspring were used because several previous studies have found little evidence of sex difference in nicotine SA [7], [39], [40]. Adolescent animals were used because we have previously shown [7] that, similar to human smokers, adolescent rats acquire nicotine self-administration at faster rates and reach higher intake levels than adults.

### Apparatus

Experiments were conducted in operant chambers (Coulbourn Instruments, Whitehall PA, USA) located in sound attenuating enclosures as described previously [7]. Briefly, one wall of each chamber was equipped with two stimulus lights located above two non-retractable levers positioned 4 cm above the grid floor. A house-light fixture was located on the opposite wall.

### Initial operant training

Male rats from each strain or F1 cross were randomly assigned to receive either nicotine or food SA. No more than two animals from the same litter were used in a group. For both groups, rats were food-deprived for 24 h on postnatal day (PN) 32, prior to being trained to press a lever to obtain food pellets (45mg, 5TUM Mlab Rodent Tablet, TestDiet). Rats received food equivalent to 10% of their body weight throughout the remainder of the experiment. This initial training terminated when rats received a minimum of 20 pellets on 2 consecutive days, usually realized within 3–4 days.

### Surgery

On PN38, each rat was implanted with a jugular catheter (constructed of PE-60 and silastic tubing), as described previously [3], [7]. Rats receiving food SA were also fitted with a jugular catheter to control for the potential effect of surgery on operant behavior. Rats recovered for 3 d and received antibiotic (0.3mg/kg enrofloxacin, Bayer). Jugular catheters were flushed daily with heparinized saline (20 IU in 0.1ml, Butler Schein).

### Nicotine SA and food rewarded behavior

Two hour SA sessions were all conducted in operant chambers during the dark phase of the light cycle. This limited access model was preferable to our 23 h access model [3] because of the large throughput of animals required in this study. Additionally, 2 h sessions were applicable to both nicotine and food reward. The house light was illuminated, signaling the beginning of each experimental session. For nicotine SA, pressing the active lever triggered the delivery of 0.03 mg/kg nicotine (50 µl/0.81 s) under a fixed-ratio (FR) 1 schedule. Infusion volumes were adjusted to accommodate different body weights. During drug delivery, the house light was extinguished and the cue light illuminated above the active lever. Each infusion was followed by a 60 s timeout, during which the house light remained off and the cue light on [41], [42]. Pressing the inactive lever was recorded, but had no programmed consequence. The house light was extinguished at the end of the session. A total of 10 consecutive daily sessions were conduced without interruption or nicotine priming. The number of parental rats used was 10, 8, 8, 10, 9, and 10 for BN, DA, F344, LEW, SHR and WKY inbred strains, respectively, while 8, 10, 11, 11, 6 and 8 F1 offspring were used for the FD, FL, FS, LB, LS, and WL.

Food rewarded behavior was conducted using a similar procedure. Rats responded for a 45 mg food pellet on an FR1 schedule with 60 s timeout during 10 consecutive daily 2 h sessions. The number of parental rats used was 9,8,11,9,7 and 9 for BN, DA, F344, LEW, SHR and WKY inbred strains, respectively, while 10, 10, 9, 9, 8, and 10 rats were used for the FD, FL, FS, LB, LS, and WL F1 offspring, respectively.

### Statistics

Data are presented as mean ± standard error of mean (SEM). The effects of strain on lever press activity and rewards (both nicotine and food) were analyzed using repeated measures ANOVA with day and lever as within subject variables. The correlations between nicotine and food were calculated for the average number of rewards earned and average number of active lever presses emitted by each strain during the last 3 d. All inbred and F1 crosses are isogenic, permitting the calculation of narrow-sense heritability from this dataset. The between-strain variance provides a measure of additive genetic variation (V_A_), while within-strain variance represents environment variability (V_E_). An estimate of narrow-sense heritability (i.e. the proportion of total phenotypic variation that is due to the *additive* effects of genes, h^2^) for nicotine or food reward was obtained using the formula: *h^2^ = V_A_/(V_A_+V_E_)* [43], which has been utilized by other groups [44], [45]. Epistasis was calculated using contrasts between an F1 and the expected mean of the two parental strains for the number of nicotine reinforcements obtained during the last 3 d [46]. Statistical analyses were performed using either R statistical package or SAS. Statistical significance was assigned when p<0.05.
